# Association Between Depressive Symptoms and Altered Heart Rate Variability in Obstructive Sleep Apnea

**DOI:** 10.3390/jcm14196978

**Published:** 2025-10-02

**Authors:** Ji Hye Shin, Min Ji Song, Ji Hyun Kim

**Affiliations:** Department of Neurology, Korea University Guro Hospital, Korea University College of Medicine, Seoul 08308, Republic of Korea; snjih@naver.com (J.H.S.); songminji1034@gmail.com (M.J.S.)

**Keywords:** depression, obstructive sleep apnea, heart rate variability

## Abstract

**Background/Objectives:** Obstructive sleep apnea (OSA) is strongly associated with cardiovascular morbidity, and depressive symptoms are common in affected individuals. Both OSA and depression have been linked to autonomic dysfunction, but the independent contribution of depressive symptoms to autonomic dysfunction in OSA remains unclear. We investigated whether depressive symptom severity is associated with autonomic function, indexed by heart-rate variability (HRV), in patients with OSA. **Methods:** We retrospectively analyzed 1713 adults with OSA at a university-affiliated sleep center from 2011 to 2024. HRV was derived from electrocardiography during polysomnography, and frequency-domain indices (natural log-transformed LF, HF, VLF, TP, and LF/HF) were computed. Depressive symptoms were assessed using the Beck Depression Inventory-II (BDI-II). Associations between BDI-II and HRV indices were evaluated using univariable and multivariable linear regressions. **Results:** In univariable regression analyses, higher BDI-II scores were significantly associated with lower HRV indices (ln LF, ln HF, ln VLF, ln TP; all *p* < 0.01). In multivariable analyses, higher BDI-II scores were independently associated with lower ln LF, ln HF, and ln TP (all *p* < 0.05), adjusting for age, sex, body mass index, hypertension, diabetes, apnea–hypopnea index, arousal index, and sleep quality. **Conclusions:** Greater depressive symptom burden is independently associated with reductions in multiple HRV indices, suggesting attenuated parasympathetic activity and autonomic dysregulation in patients with OSA. These findings support integrated management strategies that address both physiological and psychological domains in OSA and motivate longitudinal studies to test whether effective depression treatment improves HRV and mitigates long-term cardiovascular risk.

## 1. Introduction

Obstructive sleep apnea (OSA) is an independent risk factor for a range of cardiovascular and cerebrovascular diseases, including hypertension, coronary artery disease, heart failure, atrial fibrillation, stroke, and sudden cardiac death [1,2]. In addition to these physical health risks, individuals with OSA are also more likely to experience mental health disturbances, particularly depressive symptoms, with prevalence estimates ranging from 17% to 48% among this population [3]. The coexistence of OSA and depression has been shown to exacerbate clinical outcomes; for instance, one study reported that post-myocardial infarction patients with both conditions faced a substantially increased risk of mortality and recurrent infarction [4]. These findings suggest a potential interaction between depressive symptoms and OSA-related pathophysiology that may elevate cardiovascular risk.

One proposed mechanism underlying this interaction involves dysfunction of the autonomic nervous system (ANS). OSA has been consistently associated with autonomic imbalance, characterized by heightened sympathetic activity and diminished parasympathetic modulation [5,6,7,8,9,10,11]. Similarly, ANS dysregulation has been reported in individuals with mood disorders, including major depressive disorder (MDD) [12,13,14,15,16,17,18,19] and generalized anxiety disorder (GAD) [20,21,22,23,24,25]. Specifically, patients with MDD often exhibit decreased parasympathetic and increased sympathetic activity [17,18,19], whereas those with GAD show reductions in both sympathetic and parasympathetic activity [21,22,24].

Heart rate variability (HRV), a well-established and non-invasive marker of cardiac autonomic regulation, reflects the dynamic interplay between sympathetic and parasympathetic inputs to cardiac control [26]. Prior studies have examined HRV during sleep in individuals with OSA, noting that comorbid depression is associated with significant reductions in both low-frequency (LF) and high-frequency (HF) spectral components (band powers) of HRV [27,28].

More recently, studies have underscored the importance of depression in OSA. Shaw et al. demonstrated that single-lead ECG recordings during sleep can screen for MDD in OSA, supporting the feasibility of accessible, objective diagnostic tools [29]. In addition, Ditmer et al. reported that serotonergic signaling may modulate depressive symptoms and quality of life in OSA, highlighting potential neurochemical mechanisms underlying psychiatric comorbidity [30]. Taken together, these findings represent both diagnostic and mechanistic advances and reinforce the growing recognition of depression as a clinically relevant factor in OSA.

Despite the high prevalence of depressive symptoms among individuals with OSA [3], most studies used small sample sizes, and the extent to which depression contributes to ANS imbalance in OSA remains uncertain. For example, a recent study of 104 participants (34 OSA with MDD, 35 OSA without MDD, and 35 controls) reported reduced LF in OSA with MDD [27], while another study of 86 participants (40 OSA with MDD, 40 OSA without MDD, and 6 controls) found reductions in both LF and HF in OSA with MDD [28]. Although these studies offered preliminary evidence for a role of depression in ANS dysfunction in OSA, they were underpowered and limited in generalizability due to small samples. In contrast, our study included 1,713 participants, providing greater statistical power and external validity. We therefore evaluated HRV alterations in patients with OSA and tested whether depressive symptoms are associated with ANS dysfunction in this population.

## 2. Materials and Methods

### 2.1. Study Population

We conducted a retrospective study of 2811 patients with OSA who underwent overnight polysomnography (PSG) at the Korea University Guro Hospital Sleep Disorders Center between 2011 and 2024. Patients were excluded if they had a history of heart failure, angina pectoris, stroke, atrial fibrillation, or any diagnosed psychiatric disorder. Those OSA patients with comorbid sleep disorders known to influence autonomic function (e.g., narcolepsy, rapid eye movement (REM) sleep behavior disorder [31]) were further excluded. Patients in the final analysis were not taking any medications that could affect autonomic function, including parasympathomimetic medications (e.g., pilocarpine, bethanechol), anticholinergic agents (e.g., benztropine, trihexyphenidyl), or sympathomimetic drugs (e.g., isoproterenol, phenylephrine, salbutamol). OSA patients were categorized by Beck Depression Inventory-II (BDI-II) scores as nondepressed (0–13) or depressed (≥14). This study was approved by the local ethics committee of Korea University Guro Hospital (IRB No. 2025GR0322).

### 2.2. Polysomnography

Overnight PSG recording was conducted using the Embla N7000 system (Natus Medical Inc., Pleasanton, CA, USA). Electroencephalographic signals were recorded using four electrode pairs (C3-A2, C4-A1, O1-A2, and O2-A1), along with two pairs of electro-oculographic leads. Electromyographic activity was monitored from the electrodes placed on the tibialis anterior and submentalis muscles. Continuous airflow was measured using a thermistor and nasal pressure cannula, and arterial oxygen saturation was recorded via pulse oximetry. Respiratory movements were monitored using inductive plethysmographic belts placed around the chest and abdomen.

Sleep staging and respiratory event scoring were performed according to the American Academy of Sleep Medicine guidelines [32]. Sleep architecture parameters included total sleep time, sleep latency, wake after sleep onset, time in bed, sleep stage percentages (N1, N2, N3, REM), number of arousals, and sleep efficiency. Apnea was defined as a ≥90% reduction in airflow for ≥10 s, and hypopnea as a ≥30% reduction in airflow for ≥10 s accompanied by either ≥3% oxygen desaturation or an arousal [30]. The apnea–hypopnea index (AHI) was calculated as the number of apneas and hypopneas per hour of total sleep time.

### 2.3. Heart Rate Variability

Electrocardiography signals extracted from PSG were visually inspected to ensure quality and reliability. Ectopic beats and artifacts were automatically detected and removed, and only normal-to-normal beats were retained for analysis [33]. HRV was analyzed using both time-domain and frequency-domain parameters. While time-domain HRV parameters reflect overall beat-to-beat interval variability from both sympathetic and parasympathetic input, frequency-domain parameters derived using fast Fourier transform can provide more specific insights into changes in sympathetic and parasympathetic activities [26]. To more accurately assess autonomic function, we focused on frequency-domain parameters.

Spectral analysis was conducted using RemLogic software (Version 2.0; Embla Co., Broomfield, CO, USA), yielding the following frequency-domain indices: (1) LF band power (0.04–0.15 Hz), (2) HF band power (0.15–0.40 Hz), (3) very low-frequency band power (VLF; 0.0033–0.04 Hz), and (4) total power (TP). LF and HF are commonly regarded as indicators of baroreflex-mediated sympathetic activity and parasympathetic activities, respectively [31]. The LF/HF ratio serves as a marker of sympathovagal balance, with lower values reflecting parasympathetic dominance and higher values indicating sympathetic dominance due to fight-or-flight responses or parasympathetic withdrawal. VLF is thought to reflect parasympathetic tone, while TP represents overall autonomic function.

### 2.4. Questionnaires

All patients were instructed to complete self-reported symptom questionnaires. Depressive symptoms were assessed using BDI-II, with higher scores indicating greater severity [34]. Sleep quality was evaluated using the Pittsburgh Sleep Quality Index (PSQI) [35], with scores > 5 indicating poor sleep quality.

### 2.5. Statistical Analysis

Descriptive statistics were used to summarize baseline and PSG characteristics. The Kolmogorov–Smirnov test was conducted first to assess normality, revealing that all variables did not follow a normal distribution (*p* < 0.05). Therefore, natural logarithmic (ln) transformation was applied to the variables such as age, body mass index (BMI), AHI, and HRV parameters (LF, HF, VLF, TP, LF/HF). Patients were stratified by depression status based on BDI-II cutoffs, and between-group differences were assessed with two-sample *t* tests for continuous variables and chi-square tests for categorical variables.

Univariable linear regression was used to explore associations between HRV parameters and clinical variables. Multivariable linear regression was subsequently carried out to examine associations between the dependent variables (frequency-domain HRV parameters) and independent variables (demographic factors, AHI, questionnaire scores). Multicollinearity among independent variables was evaluated using variance inflation factors, with a threshold of <5 for inclusion. All statistical analyses were conducted using SPSS software (version 29.0; IBM Corp., Armonk, NY, USA), and the results were considered statistically significant at *p* < 0.05.

## 3. Results

**Figure 1:**
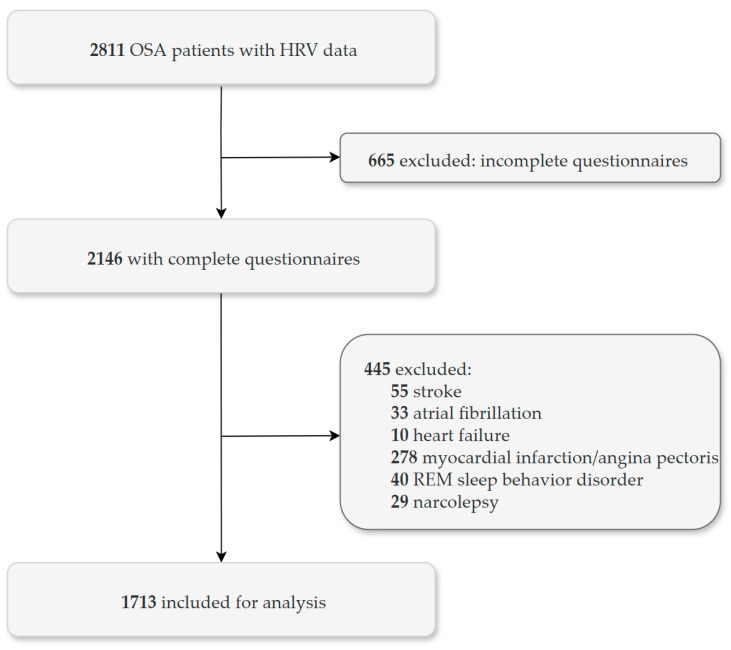
Flow diagram depicting the patient selection process. Abbreviations: HRV, heart rate variability; OSA, obstructive sleep apnea.

A total of 2811 OSA patients with available HRV data were screened for inclusion in this study. Of these, 1098 patients were excluded for the following reasons: incomplete questionnaire responses (*n* = 665), heart failure (*n* = 10), angina pectoris (*n* = 278), stroke (*n* = 55), atrial fibrillation (*n* = 33), narcolepsy (*n* = 29), or REM sleep behavior disorder (*n* = 40). The final analytic sample comprised 1713 patients, including 1278 males and 435 females (Figure 1). Demographics, clinical characteristics, sleep questionnaire scores, and PSG data of the study population are summarized in Table 1. Compared with nondepressed OSA group (*n* = 1018), the depressed OSA group (*n* = 695) had a lower proportion of males (*p* < 0.001), was older (*p* = 0.012), and had a smaller neck circumference (*p* < 0.001). Questionnaire scores indicated a greater symptom burden in the depressed group than in the nondepressed group (Epworth sleepiness scale, insomnia severity index, and PSQI; all *p* < 0.001). Among PSG parameters, the depressed group had shorter total sleep time and lower sleep efficiency compared with the nondepressed group (both *p* < 0.001), while two groups did not differ in AHI and arousal index. In frequency-domain HRV analyses, LF, HF, VLF, and TP were lower in the depressed group than in the nondepressed group (all *p* < 0.05), whereas the LF/HF ratio did not differ between groups.

The results of univariable linear regression analysis examining associations between independent variables and HRV parameters are presented in Table 2. ln LF was significantly higher in males (*p* < 0.001) and positively associated with BMI (*p* < 0.05), AHI (*p* < 0.001), and arousal index (*p* < 0.001). ln LF was negatively associated with age, diabetes mellitus, and BDI-II scores (all *p* < 0.001). ln HF was significantly higher in males (*p* < 0.001), and negatively associated with age, hypertension, diabetes mellitus, arousal index (all *p* < 0.001), and BDI-II scores (*p* < 0.01). ln VLF was significantly higher in males (*p* < 0.001), and negatively associated with age (*p* < 0.05), diabetes mellitus (*p* < 0.01), and BDI-II scores (*p* < 0.01). Positive associations were also observed with BMI (*p* < 0.001), AHI (*p* < 0.001), and arousal index (*p* < 0.01). ln TP was higher in males (*p* < 0.001), and negatively associated with age, diabetes mellitus, and BDI-II scores (all *p* < 0.001). It was positively associated with BMI (*p* < 0.001), AHI (*p* < 0.001), and arousal index (*p* < 0.05). ln LF/HF ratio was higher in males (*p* < 0.001), and positively associated with age (*p* < 0.01), BMI (*p* < 0.01), hypertension (*p* < 0.01), AHI (*p* < 0.001), and arousal index (*p* < 0.001). BDI-II scores showed consistent inverse associations with frequency-domain HRV indices, although the strength of these associations was modest. The strongest associations were observed for ln LF and ln TP (both *p* < 0.001), with weaker associations for ln HF and ln VLF (both *p* < 0.01). No significant association was observed for the LF/HF ratio.

The results of multivariable linear regression analysis are presented in Table 3 and confirmed the findings of univariable regression analysis. Higher BDI-II scores were significantly associated with lower values of ln LF (β = –0.005, *p* < 0.05), ln HF (β = –0.004, *p* < 0.05), and ln TP (β = –0.004, *p* < 0.05). No significant associations were observed between BDI-II scores and either ln VLF or ln LF/HF.

## 4. Discussion

This study examined the association between HRV and depressive symptoms in a large cohort of OSA patients. The main findings are that higher BDI-II scores were significantly associated with lower HRV indices, including ln LF, ln HF, ln VLF, and ln TP, indicating diminished overall autonomic activity. In multivariable linear regression, higher BDI-II scores remained independently associated with reduced ln LF, ln HF, and ln TP, supporting an independent link between depressive symptom burden and attenuated ANS function in OSA.

Our findings align with prior literature demonstrating a relationship between depression and autonomic dysregulation characterized predominantly by reduced parasympathetic activity [12,13,14,16,36]. For example, adolescent females with MDD exhibited significantly lower LF and HF than healthy controls [16]. Similarly, reviews report that both clinical and non-clinical young populations with depression or anxiety show lower HRV relative to controls [12]. In a longitudinal cohort of unmedicated patients with depression, HF was reduced at baseline and increased following antidepressant treatment alongside symptomatic improvement, implicating impaired parasympathetic regulation in depression [36]. A meta-analysis of four studies reported a moderate effect size indicating lower HF among children and adolescents with clinical depression than controls [13]. Notably, some work has observed a different pattern, with consistently lower HF but higher LF in patient with depression compared with controls, contrasting studies that reported decreases in both components [14]. Together, these data suggest that, although reduced parasympathetic activity (lower HF) is a robust finding, LF behavior may vary across populations and analytic approaches.

Assessing ANS function offers a useful framework for understanding emotional symptoms. Increased sympathetic activity is associated with emotional instability, irritability, and fatigue, whereas augmented parasympathetic activity corresponds to a calmer mental state [37]. In HRV analysis, reduced HF power has been linked to stress, panic, anxiety, and worry [38]. Several studies have shown a consistent association between anxiety and reduced HF, again reflecting diminished parasympathetic tone. In a Taiwanese case–control study, patients with GAD demonstrated lower LF and HF—most prominently among those with comorbid MDD—than controls [24]. Symptom severity of anxiety and depression correlated with both HF and LF, indicating a strong relationship between emotional dysregulation and autonomic imbalance [24]. Across resting, relaxation, and worry conditions, individuals with GAD consistently exhibited reduced HF relative to non-anxious controls [22], and a meta-analysis found significantly lower HF across anxiety disorders irrespective of specific diagnosis [21]. These convergent findings reinforce the central role of parasympathetic activity regulation in affective symptomatology.

The precise mechanisms linking mood disturbances and autonomic dysfunction remain incompletely defined. One hypothesis is autonomic rigidity, characterized by persistently low parasympathetic activity, may reduce physiological flexibility and impair adaptive behavior [25]. Such rigidity may stem from disrupted interaction between parasympathetic and sympathetic branches, leading to vagal brake dysfunction and reduced HRV [39]. Similarly, OSA is known to be associated with altered HRV patterns reflecting shifts in autonomic balance. Polyvagal theory provides a complementary framework for understanding the relationship between ANS dysregulation and affective symptoms. The vagus nerve, particularly its ventral branch, has been implicated in regulating physiological state and socio-emotional behavior through a “social engagement system” that promotes calm states and emotional regulation during social interaction [40]. When vagal tone is diminished, the social engagement system may be blunted, predisposing to emotional dysregulation and depressive symptoms [41]. Our results are consistent with this hypothesis: individuals with higher BDI-II scores exhibited lower parasympathetic activity across multiple HRV indices.

Studies of cardiopulmonary phase synchronization in OSA further underscore the interplay between ANS and mood disorders. An HRV-based study reported significantly higher cardiopulmonary phase synchronization in OSA patients with comorbid MDD than in those without. This finding suggests that comorbid depression in OSA may be associated with lower sympathetic drive and/or greater vagal modulation, facilitating tighter coupling between respiratory sinus arrhythmia and respiration (i.e., increased phase-locking) [28]. In our study, depressive symptoms were inversely associated with LF power, a finding consistent with these mechanisms, although alternative or additional processes likely contribute. Under resting condition, the LF band (0.04–0.15 Hz) primarily reflects baroreflex-mediated oscillations. Because the sympathetic nervous system does not generate rhythms above 0.1 Hz and parasympathetic influences extend into the LF range to 0.05 Hz [38], LF power is generally interpreted as an index of integrated autonomic (baroreflex) modulation rather than a purely sympathetic marker.

Our results showed that HRV parameters were also associated with demographic and clinical factors, including age, sex, diabetes mellitus, and OSA severity. Notably, AHI showed a positive association with several HRV components, particularly ln LF and ln TP. This seemingly paradoxical pattern—greater OSA severity accompanying increased HRV—likely reflects enhanced sympathetic activity driven by apneic cycles, arousals, intermittent hypoxemia, and baroreflex engagement, rather than “healthier” autonomic function [8]. By contrast, the inverse association between depressive symptoms and HRV suggests a distinct autonomic signature characterized by predominant parasympathetic withdrawal rather than isolated sympathetic overactivity. By contrast, the inverse association between depressive symptoms and HRV suggests a distinct autonomic signature characterized by predominant parasympathetic withdrawal rather than isolated sympathetic overactivity.

This study has several potential limitations. First, the retrospective and single-center design of the study limits causal inference. Second, potential selection bias from a university-affiliated hospital cohort may reduce the generalizability of the findings. Moreover, the single-center Korean cohort may limit the generalizability of the findings to more diverse populations. The exclusion of patients with comorbidities or medications affecting ANS function may further limit the generalizability of our findings to the broader OSA population. Third, sex differences may also confound mood symptoms in OSA, as OSA is more prevalent in men whereas depression is more common in women. Women with OSA are reported to have markedly higher odds of depressive symptoms even after adjustments for age, BMI, and OSA indices [42]. Lastly, HRV was derived from entire overnight PSG without excluding arousals and apneas, which may influence HRV estimates. Although this approach captures the overall autonomic profile throughout the night, it may increase variability due to sleep stage transitions and respiratory events. Future work should incorporate standardized artifact handling, sleep-stage stratification, and prospective design. Moreover, HRV assessment in short, standardized epochs during wakefulness and during autonomic function tests (e.g., active standing/orthostasis, paced deep breathing), may further improve reproducibility and physiological interpretability.

Beyond the observed association between depressive symptoms and HRV, OSA is increasingly recognized as a multisystem disorder with comorbidities across cardiovascular, cerebrovascular, metabolic, neuropsychiatric, and even oncologic domains [43]. Recent reviews highlight that recurrent hypoxemia and sleep fragmentation exert pleiotropic effects—contributing to autonomic dysregulation, systemic inflammation, oxidative stress, and metabolic disturbances—which together may heighten psychiatric vulnerability [43]. These insights support an integrative clinical approach in which depression screening accompanies the evaluation of cardiometabolic and neurocognitive complications. Such multidimensional strategies may improve long-term outcomes by targeting shared pathophysiological pathways linking physical and mental health.

From a clinical perspective, the association between depressive symptoms and reduced HRV in OSA suggests that integrated management of OSA and comorbid depression may yield meaningful benefits. Effective treatment of depressive symptoms could improve adherence to positive airway pressure therapy, mitigate autonomic dysfunction, and ultimately reduce adverse cardiovascular and cerebrovascular outcomes. Future interventional studies are needed to determine whether combined treatment strategies translate into measurable improvements in psychological well-being and cardiometabolic health.

**Figure 2:**
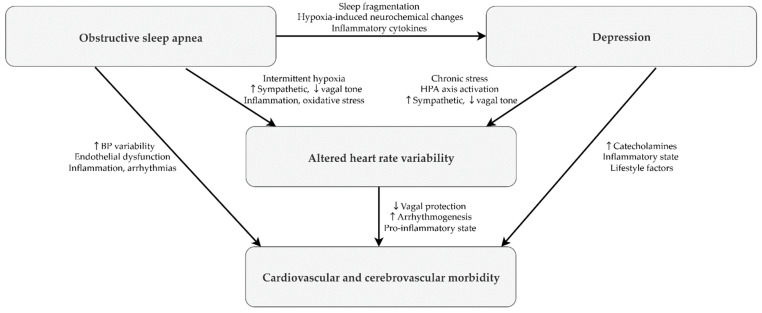
Conceptual diagram illustrating the proposed mechanistic links between OSA, depression, altered heart rate variability, and cardiovascular/cerebrovascular morbidity. Arrows denote hypothesized causal pathways. OSA contributes to depression through sleep fragmentation, hypoxia, and inflammatory activation, and affects HRV via intermittent hypoxia, autonomic imbalance, inflammation, and oxidative stress. Depression further modifies HRV through chronic stress, HPA axis activation, and autonomic dysregulation. Altered HRV, characterized by reduced vagal protection, arrhythmogenesis, and a pro-inflammatory state, ultimately increases the risk of cardiovascular and cerebrovascular morbidity.

In summary, our findings emphasize that depressive symptoms were independently associated with reductions in multiple HRV indices, supporting the hypothesis that mood disturbances may exacerbate autonomic instability in patients with OSA. Clinicians should integrate routine screening for depressive symptoms into OSA evaluations and adopt multidimensional management strategies that address both physiological and psychological domains. Interventions such as cognitive-behavioral therapy and evidence-based pharmacotherapy for depression may help mitigate autonomic dysfunction. Future prospective studies are warranted to test whether effective treatment of depressive symptoms leads to sustained improvements in HRV and mitigates long-term cardiovascular outcomes among individuals with OSA. Figure 2 provides a conceptual framework outlining hypothesized pathways linking OSA, depression, HRV alterations, and cardiovascular/cerebrovascular morbidity. This model is intended to facilitate interpretation and to motivate further research [7,8,44,45,46,47,48,49,50,51].

## 5. Conclusions

Depressive symptoms in OSA are independently associated with reductions in HRV indices, reflecting ANS dysfunction. Integrating depression screening and evidence-based treatment into OSA care may improve psychological outcomes and help mitigate autonomic dysregulation. Given the established links between ANS dysfunction and cardiovascular and cerebrovascular morbidity, prospective longitudinal studies are warranted to determine whether targeted treatment of depressive symptoms improves HRV and long-term clinical outcomes in patients with OSA.

## Figures and Tables

**Table 1:** Baseline clinical characteristics of the study population.

	Depressed OSA (*n* = 695)	Nondepressed OSA (*n* = 1018)	*p*-Value
**Baseline characteristics**			
Male, *n* (%)	456 (65.6%)	822 (80.7%)	<0.001
Age (years)	51.3 ± 14.0	49.6 ± 13.1	0.012
BMI (kg/m^2^)	27.8 ± 5.0	27.7 ± 4.3	0.842
Hypertension, *n* (%)	326 (46.9%)	498 (48.9%)	0.413
Diabetes mellitus, *n* (%)	109 (15.7%)	149 (14.6%)	0.552
Smoking, *n* (%)	171 (24.6%)	87 (8.5%)	<0.001
Neck circumference	38.2 ± 4.0	39.1 ± 3.6	<0.001
Epworth sleepiness scale	8.4 ± 4.9	7.2 ± 4.1	<0.001
Insomnia severity index	15.3 ± 6.3	10.1 ± 6.0	<0.001
Beck Depression Inventory-II	21.8 ± 6.5	7.1 ± 3.8	<0.001
PSQI	10.2 ± 4.2	7.0 ± 3.6	<0.001
**PSG parameters**			
Total sleep time (min)	335.6 ± 63.7	347.9 ± 50.8	<0.001
Sleep efficiency (%)	80.5 ± 15.2	84.1 ± 12.2	<0.001
WASO (min)	65.7 ± 55.8	54.3 ± 46.0	<0.001
N1 (min)	111.9 ± 53.1	115.7 ± 49.3	0.128
N2 (min)	126.5 ± 53.9	131.1 ± 48.7	0.069
N3 (min)	13.1 ± 22.0	13.1 ± 22.7	0.994
REM (min)	84.2 ± 37.3	88.1 ± 32.1	0.026
Apnea–hypopnea index	28.5 ± 23.6	30.1 ± 23.0	0.162
Arousal index	45.6 ± 22.5	45.4 ± 21.6	0.880
LF (ms^2^)	10,744.7 ± 7171.2	11,746.4 ± 7725.7	0.007
HF (ms^2^)	5238.2 ± 2908.8	5576.3 ± 3028.9	0.021
VLF (ms^2^)	16,746.6 ± 11,169.2	18,218.3 ± 12,551.1	0.011
TP (ms^2^)	33,557.8 ± 18,391.2	36,420.7 ± 20,139.7	0.003
LF/HF	2.5 ± 2.5	2.8 ± 8.6	0.347

Abbreviations: BMI, body mass index; HF, high frequency; LF, low frequency; PSQI, Pittsburgh Sleep Quality Index; REM, Rapid Eye Movement sleep, TP, total power; VLF, very low frequency; WASO, waking after sleep onset. Data are presented as means ± standard deviations. *P*-values were calculated using two-sample *t* tests for continuous variables and chi-square tests for categorical variables. Groups were classified by BDI-II (0–13 = no depression; ≥14 = depression).

**Table 2:** Results of univariable regression analyses.

Variables	ln LF	ln HF	ln VLF	ln TP	ln LF/HF
Age	−0.46 *** (−0.56, −0.36)	−0.61 *** (−0.71, −0.52)	−0.15 * (−0.26, −0.04)	−0.31 *** (−0.39, −0.22)	0.16 ** (0.05, 0.26)
Male sex	0.50 *** (0.43, 0.57)	0.12 *** (0.05, 0.19)	0.38 *** (0.30, 0.46)	0.37 *** (0.31, 0.43)	0.38 *** (0.31, 0.45)
BMI	0.26 * (0.06, 0.46)		0.46 *** (0.23, 0.68)	0.32 *** (0.15, 0.49)	0.30 ** (0.10, 0.50)
Hypertension		−0.15 *** (−0.20, −0.09)	0.06 (−0.01, 0.13)	0.00 (−0.05, 0.05)	0.09 ** (0.03, 0.15)
Diabetes mellitus	−0.24 *** (−0.33, −0.15)	−0.29 *** (−0.37, −0.20)	−0.16 ** (−0.25, −0.06)	−0.19 *** (−0.26, −0.11)	
AHI	0.14 *** (0.10, 0.18)	−0.09 *** (−0.13, −0.05)	0.17 *** (0.12, 0.21)	0.12 *** (0.08, 0.15)	0.23 *** (0.19, 0.27)
Arousal index	0.003 *** (0.002, 0.005)	−0.005 *** (−0.006, −0.003)	0.002 ** (0.001, 0.004)	0.001 * (0.000, 0.003)	0.008 *** (0.006, 0.009)
BDI-II	−0.008 *** (−0.012, −0.005)	−0.005 ** (−0.008, −0.001)	−0.006 ** (−0.010, −0.002)	−0.006 *** (−0.009, −0.003)	
PSQI					

Values are expressed as unstandardized beta coefficients (95% Confidence Interval); * *p* < 0.05, ** *p* < 0.01, *** *p* < 0.001. Age, BMI, AHI, LF, HF, VLF, TP and LF/HF were transformed using the natural logarithm. Abbreviations: AHI, apnea–hypopnea index; BDI-II, Beck Depression Inventory-II; BMI, body mass index; HF, high frequency; LF, low frequency; PSQI, Pittsburgh Sleep Quality Index; TP, total power; VLF, very low frequency. Columns without correlation data were removed for clarity.

**Table 3:** Results of multivariable linear regression analysis for variables.

Variables	ln LF	ln HF	ln VLF	ln TP	ln LF/HF
Age	−0.274 *** (−0.383, −0.166)	−0.587 *** (−0.689, −0.486)	0.023 (−0.102, 0.147)	−0.162 *** (−0.254, −0.069)	0.313 *** (0.205, 0.420)
Male sex	0.382 *** (0.307, 0.457)	0.010 (−0.061, 0.080)	0.321 *** (0.234, 0.407)	0.280 *** (0.216, 0.344)	0.372 *** (0.298, 0.447)
BMI	−0.054 (−0.279, 0.170)	−0.065 (−0.276, 0.145)	0.237 (−0.021, 0.495)	0.094 (−0.097, 0.286)	0.011 (−0.212, 0.234)
Hypertension	−0.024 (−0.090, 0.041)	−0.011 (−0.072, 0.050)	0.030 (−0.046, 0.105)	0.011 (−0.045, 0.067)	−0.013 (−0.078, 0.052)
Diabetes mellitus	−0.183 *** (−0.269, −0.097)	−0.192 *** (−0.273, −0.111)	−0.176 *** (−0.276, −0.077)	−0.166 *** (−0.240, −0.092)	0.009 (−0.077, 0.095)
AHI	0.106 *** (0.051, 0.160)	0.007 (−0.044, 0.058)	0.145 *** (0.082, 0.208)	0.110 *** (0.063, 0.156)	0.099 *** (0.044, 0.153)
Arousal index	0.000 (−0.002, 0.002)	−0.005 *** (−0.006, 0.003)	−0.002 * (−0.004, 0.000)	−0.002 * (−0.003, 0.000)	0.005 *** (0.003, 0.006)
BDI-II	−0.005 * (−0.008, −0.001)	−0.004 * (−0.007, 0.000)	−0.003 (−0.008, 0.001)	−0.004 * (−0.007, −0.001)	−0.001 (−0.004, 0.003)
PSQI	0.096 ** (0.026, 0.167)	0.040 (−0.026, 0.106)	0.125 ** (0.044, 0.206)	0.083 ** (0.022, 0.143)	0.056 (−0.013, 0.126)

Values are expressed as unstandardized beta coefficients (95% Confidence Intervals); * *p* < 0.05, ** *p* < 0.01, *** *p* < 0.001. Age, BMI, AHI, LF, HF, VLF, TP, and LF/HF were transformed using the natural logarithm. Abbreviations: AHI, apnea–hypopnea index; BDI-II, Beck Depression Inventory-II; BMI, body mass index; HF, high frequency; LF, low frequency; PSQI, Pittsburgh Sleep Quality Index; TP, total power; VLF, very low frequency.

## Data Availability

The dataset used and analyzed in the current study is available from the corresponding author on reasonable request.

## Abbreviations

The following abbreviations are used in this manuscript:

OSA	Obstructive sleep apnea
HRV	Heart rate variability
PSG	Polysomnography
REM	Rapid eye movement
AHI	Apnea–hypopnea index
LF	Low-frequency band power
HF	High-frequency band power
VLF	Very low-frequency band power
TP	Total power spectrum
BDI-II	Beck Depression Inventory II
